# Plant-derived extracellular vesicles in facial aesthetics

**DOI:** 10.20517/evcna.2025.43

**Published:** 2025-10-17

**Authors:** Nan Yang, Yucui Wang, Hailong Liu, Tianhao Li, Ming Lu, Rong Lu

**Affiliations:** ^1^Marine College, Shandong University, Weihai 264209, Shandong, China.; ^2^Shandong Feihua Brand Management Co., Ltd., Zaozhuang 277599, Shandong, China.

**Keywords:** Plant-derived extracellular vesicles, facial aesthetics, anti-scarring, anti-aging, anti-pigmentation

## Abstract

In the rapidly developing field of skin care, non-surgical facial aesthetics are becoming increasingly favored by consumers. Plant-derived extracellular vesicles (PDEVs) have attracted much attention due to their low toxicity, cellular communication function, and ability to carry bioactive molecules, including proteins, lipids, nucleic acids, and small molecules with pharmacological activities. Recent *in vitro* studies have shown that PDEVs enhance the transdermal delivery of drugs and improve skin condition, suggesting promising applications in facial aesthetics. In this review, we provide a comprehensive overview of the application of PDEVs in anti-scarring, anti-aging, and anti-pigmentation therapies. We also discuss current limitations in their application and potential solutions to address these challenges. In conclusion, this review analyzes the roles and mechanisms of PDEVs in facial aesthetics and aims to support their future clinical application.

## INTRODUCTION

Since ancient times, humans have pursued ideals of beauty. With societal development and technological advancement, aesthetic preferences have evolved, and skin concerns such as wrinkles, aging, and hyperpigmentation have gradually become the focus of attention. Skin problems not only affect appearance but also reflect people's lifestyles and health conditions^[1]^. Facial aesthetics encompasses techniques designed to address such skin issues, thereby enhancing personal appearance and self-confidence. Compared with plastic surgery, non-surgical medical aesthetics are less invasive and involve shorter recovery times. These advantages are driving their growing adoption^[2]^. Traditional non-surgical methods primarily include energy-based devices and injectables. Energy-based devices, such as lasers, intense pulsed light, and radiofrequency devices, stimulate collagen remodeling and demonstrate significant therapeutic efficacy in skin rejuvenation^[3,4]^. However, these modalities are associated with risks of thermal injury, post-inflammatory hyperpigmentation, and scarring^[5]^. Injectable treatments, including botulinum toxin type A and dermal fillers (e.g., hyaluronic acid, poly-L-lactic acid), are used to reduce wrinkles and improve skin firmness. Nevertheless, they may result in adverse effects such as edema and facial asymmetry^[6]^.

**Figure 1:**
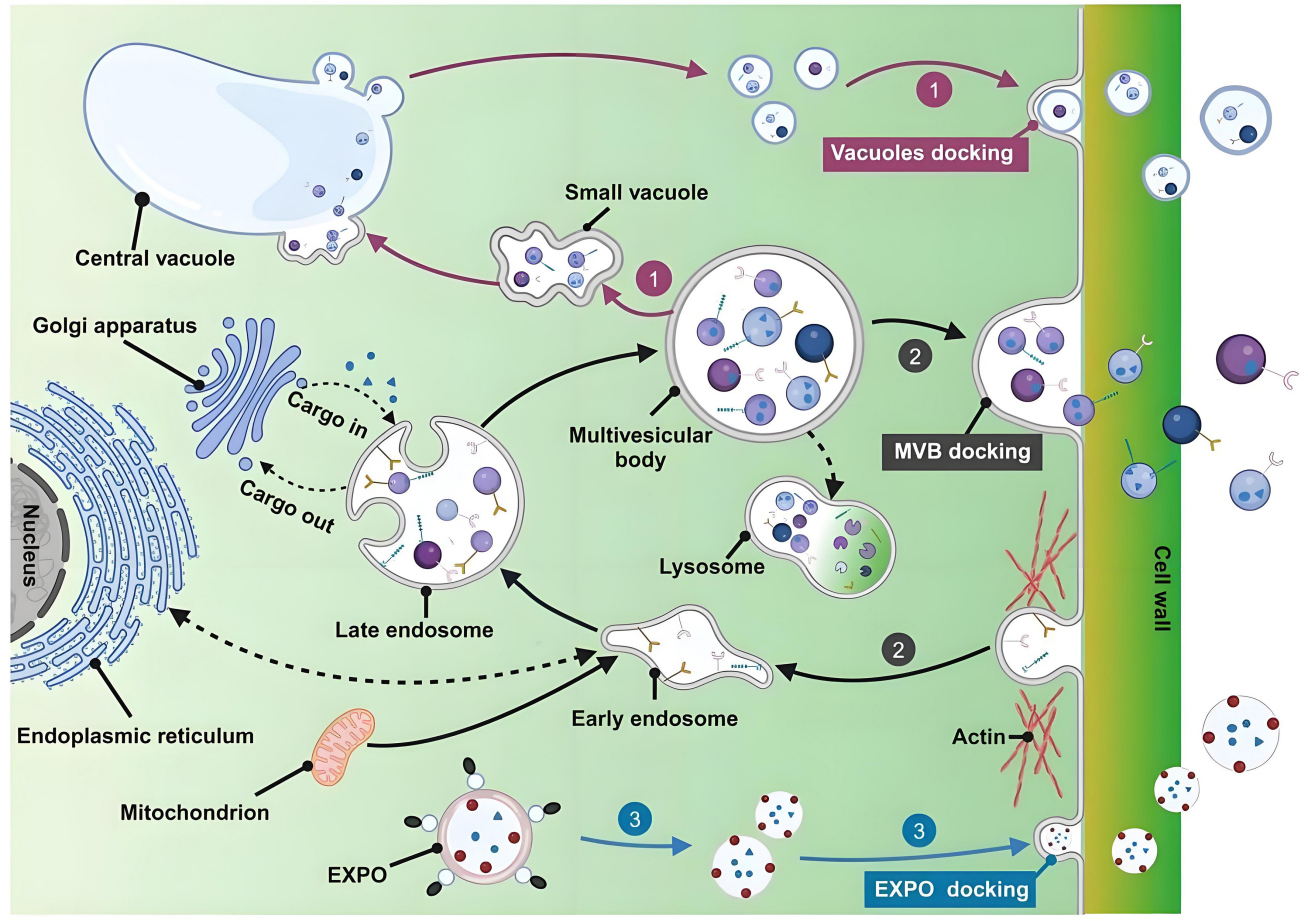
Biogenesis pathway of PDEVs. (1) Vacuolar pathway: small vacuoles in plant cells fuse to form the central vacuole, which subsequently fuses with the PM to release EVs; (2) MVBs pathway; (3) EXPO pathway: double-membrane EXPO mediates exocytosis from the cytoplasm to the cell wall. This figure is quoted with permission from Zhao *et al*.^[10]^. PDEVs: Plant-derived extracellular vesicles; PM: plasma membrane; MVBs: multivesicular bodies; EXPO: exocyst-positive organelle.

In recent years, nanotechnology has gained significant attention in the cosmetic industry due to its enhanced skin permeability, ability to protect active ingredients from degradation, improved stability, and capacity for controlled and sustained release^[7]^. Multiple nanocarriers, including liposomes, ethosomes, solid lipid nanoparticles, nanocapsules, dendrimers, nanocrystals, and niosomes, are utilized in the formulation of cosmetics and cosmeceuticals^[8]^. However, concerns regarding the potential toxicity associated with nanoparticle penetration have prompted the search for safer alternatives. Extracellular vesicles (EVs) represent a promising option for facial aesthetic applications owing to their high biocompatibility. EVs are 30-1,000 nm vesicular structures secreted by cells, including exosomes, microvesicles, and apoptotic bodies^[9]^. While EVs are universally secreted by almost all living cells, those derived from plants (PDEVs) are of particular interest in cosmetics. PDEVs are formed through the inward budding of the plasma membrane (PM), resulting in early endosomes that mature into multivesicular bodies (MVBs). These MVBs encapsulate intraluminal vesicles (ILVs), which are released into the extracellular space as exosomes upon fusion of MVBs with the PM. Moreover, PDEVs are also released via the exocyst-positive organelles (EXPO) pathway and the vacuole pathway [Figure 1]^[10]^. Compared to animal-derived exosomes, PDEVs exhibit lower immunogenicity mainly due to the lack of animal-specific antigens^[11]^. Additionally, the unique membrane architecture of PDEVs facilitates their penetration through the skin barrier. For instance, *Leontopodium alpinum* exosomes significantly improve the skin permeability of acetyl hexapeptide-8 compared to its free form, enabling targeted delivery to the dermis for wrinkle reduction^[12]^. Similarly, cucumber-derived exosome-like vesicles have been shown to double the skin permeation efficiency of lipophilic bioactive compounds^[13]^. Furthermore, PDEVs possess multifunctional properties such as antioxidant, anti-inflammatory, and tissue regenerative activities^[14-16]^. These properties enable a comprehensive mechanism for skin rejuvenation, which operates distinctly from conventional methods that depend solely on tissue filling or collagen stimulation. Therefore, PDEVs demonstrate considerable potential as an innovative and advantageous strategy in facial aesthetics.

This review explores the potential of PDEVs in facial aesthetics, with a specific focus on their roles in anti-scarring, anti-aging, and skin hyperpigmentation reduction. Furthermore, the challenges and solution strategies associated with PDEV-based therapies are discussed.

## APPLICATION OF PDEVS IN FACIAL AESTHETICS

Scarring, skin aging, and hyperpigmentation are among the most frequently encountered concerns in facial aesthetics. As an emerging nanoscale delivery system, PDEVs demonstrate significant potential in facial aesthetics due to their nanoscale structure, high biocompatibility, and low immunogenicity. The anti-scarring, anti-aging, and anti-pigmentation effects of PDEVs are summarized in Table 1.

**Table 1:** PDEVs in promoting anti-scarring, anti-aging, and anti-pigmentation effects

**Effect**	**Mechanism**	**Source of representative PDEVs**	**Reference**
Anti-scarring	Inhibition of inflammatory responses Regulation of collagen biosynthesis	Cabbage, *Solanum nigrum* L. berries, papaya fruit, apple, pomegranate, lemon, *Beta vulgaris*, aloe vera peel, rose stem cell	[17-25]
Anti-aging	Inhibition of ROS synthesis Enhancement of antioxidant capacity Promotion of collagen synthesis	Ginseng root, golden cherry, *Aloe Vera* gel and rind, glucoraphanin-enriched kale, ginseng, green tea, *Ecklonia cava*, *Olea europaea* leaf, apple, *Polygonum multiflorum*, balloon flower root	[26-35]
Anti-pigmentation	Inhibition of melanogenesis	Yam bean, *Atractylodes lancea*, *Panax ginseng*, *Ecklonia cava*, *Dendropanax morbifera*, *Codium fragile* and *Sargassum fusiforme*, *Centella asiatica*, rose stem cell	[36-43]

PDEVs: Plant-derived extracellular vesicles; ROS: reactive oxygen species.

### Anti-scarring

#### Scar formation

The formation of scars is a complex biological process that represents a pathological outcome of dysregulated repair following skin injury^[44]^. When the skin is traumatized, platelets rapidly aggregate and activate the coagulation cascade, forming a fibrin clot while releasing numerous growth factors and cytokines^[45]^. These signaling molecules stimulate the migration of surrounding keratinocytes to seal the wound and recruit immune cells to clear necrotic tissue. Macrophages play a key role in transitioning wound healing from the inflammatory to the proliferative phase. Initially, M1 macrophages secrete pro-inflammatory factors such as interleukin-1β, IL-6, and tumor necrosis factor-α (TNF-α) to eliminate necrotic tissue. However, excessive activation may amplify inflammation and promote fibrosis^[46]^. Subsequently, M2 macrophages facilitate extracellular matrix (ECM) synthesis and tissue repair, yet persistent M2 activation can contribute to pathological scarring^[47]^. Therefore, persistent excessive inflammation impairs wound healing and is associated with scar formation.

As wound healing progresses, fibroblasts synthesize ECM components such as collagen, fibronectin, and proteoglycans to repair damaged tissue. In normal wound healing, transforming growth factor-β (TGF-β) stimulates the differentiation of fibroblasts into myofibroblasts^[48]^. These cells express α-smooth muscle actin (α-SMA) and function to mediate wound contraction and produce ECM, predominantly collagen^[49]^. Subsequently, most myofibroblasts undergo apoptosis. Concurrently, matrix metalloproteinases (MMPs) facilitate the replacement of type III collagen with type I collagen, enabling ECM remodeling and the restoration of normal tissue architecture^[50]^. However, excessive inflammatory signaling leads to sustained TGF-β expression, which prolongs myofibroblast survival and promotes pathological collagen accumulation^[47]^. Furthermore, TGF-β suppresses the expression of MMP, thereby promoting abnormal collagen deposition and the formation of a disorganized ECM network, which ultimately drives scar tissue formation^[51]^. Therefore, inhibiting fibroblast-to-myofibroblast differentiation and balancing the type I/III collagen ratios may contribute to scar-free skin repair^[52]^.

#### PDEVs reduce scar formation

At present, therapeutic approaches for scar management include surgery, laser therapy, corticosteroids (injections, tapes, or ointments), and compression therapy^[53]^. Evidence suggests that PDEVs promote wound healing [Table 2], and a well-organized healing process is crucial for minimizing scar formation. PDEVs primarily attenuate scarring by suppressing inflammatory responses and modulating collagen synthesis and deposition.

**Table 2:** PDEVs in promoting wound healing

**Source of PDEVs**	**Model**	**Mechanism**	**Reference**
Coriander	*In vitro*: HaCaT *In vivo*: full-thickness wound in ICR mice	↑ cell migration ↑ IL-10 ↓ MMP-13, TNF-α	[54]
Tomato	*In vitro*: HUKE, NIH-3T3 mouse fibroblasts	↑ cell migration	[55]
*Dendrobium*	*In vivo*: total dermal excisional wound in C57BL/6J mice	↓ IL-1β	[56]
Turmeric	*In vitro*: L929 mouse fibroblasts, RAW264.7 cells *In vivo*: full-thickness wound in C57BL/6J mice	↑ cell proliferation and migration ↑ collagen I, IL-10 ↓ IL-1β, IL-6, TNF-α	[57]
*Physalis peruviana*	*In vitro*: HDF	↑ cell proliferation and migration ↑ collagen I ↓ MMP-1	[58]
Ginseng	*In vitro*: HaCaT, HUVEC *In vivo*: full-thickness skin excisional wound in mice	↑ cell proliferation and migration ↑ angiogenesis, MMP-1, fibronectin-1, elastin-1, collagen I, TGF-β ↓ iNOS, COX-2, and NF-κB	[59]
Pomegranate	*In vitro*: THP-1, Caco-2 cells	↑ cell migration ↓ NF-κB	[21]
*Aloe saponaria*	*In vitro*: HDF, HUVEC, RAW264.7 cells	↑ cell proliferation and migration ↑ angiogenesis ↓ IL-1β, IL-6	[60]
*Aloe vera* peels	*In vitro*: HaCaT, HDF	↑ cell migration	[61]
Grapefruit	*In vitro*: HaCaT, HUVEC	↑ cell proliferation and migration ↑ angiogenesis	[62]
Wheat	*In vitro*: HDF, HUVEC, HaCaT	↑ cell proliferation and migration ↑ angiogenesis, collagen I	[63]

↑ Represents upregulation; ↓ represents downregulation. PDEVs: Plant-derived extracellular vesicles; HaCaT: human keratinocyte cell line; IL: interleukin; ICR: Institute of Cancer Research; MMP: matrix metalloproteinase; TNF-α: tumor necrosis factor-α; HUKE: human keratinocyte; NIH-3T3: mouse embryonic fibroblast cell line; IL-1β: interleukin-1 beta; L929: mouse fibroblast cell line; RAW264.7: mouse monocytic macrophage leukemia cells; HDF: human dermal fibroblasts; HUVEC: human umbilical vein endothelial cells; TGF-β: transforming growth factor-β; iNOS: inducible nitric oxide synthase; COX: cyclooxygenase; NF-κB: nuclear factor kappa B; THP-1: human myeloid leukemia mononuclear cells; Caco-2: human colorectal adenocarcinoma cells.

Chronic and excessive inflammation is not conducive to wound healing and promotes scar formation^[64]^. The nuclear factor kappa B (NF-κB) signaling pathway is a key regulator of inflammatory and immune responses and plays a central role in this process^[65]^. Research indicates that PDEVs can attenuate inflammatory responses. For example, cabbage-derived exosome-like nanovesicles and EVs from *Solanum nigrum* L. berries exhibited anti-inflammatory properties by downregulating the expression of pro-inflammatory factors, including IL-1 and IL-6^[17,18]^. Similarly, Iriawati *et al*. found that papaya fruit-derived EVs suppressed pro-inflammatory cytokine expression and upregulated anti-inflammatory cytokines such as IL-10^[19]^. Consistent with these findings, EVs from apple and pomegranate demonstrated anti-inflammatory activity through the downregulation of the NF-κB signaling pathway^[20,21]^. In addition, lemon-derived EVs were found to suppress the extracellular signal-regulated kinase (ERK)/NF-κB signaling pathway, potentially inhibiting scar formation^[22]^. In this context, ERK acts as a molecular switch regulating the balance between tissue regeneration and scar development^[66]^. Collectively, these studies indicate that PDEVs function as inflammatory response modulators and represent promising anti-inflammatory agents for preventing scar formation.

**Figure 2:**
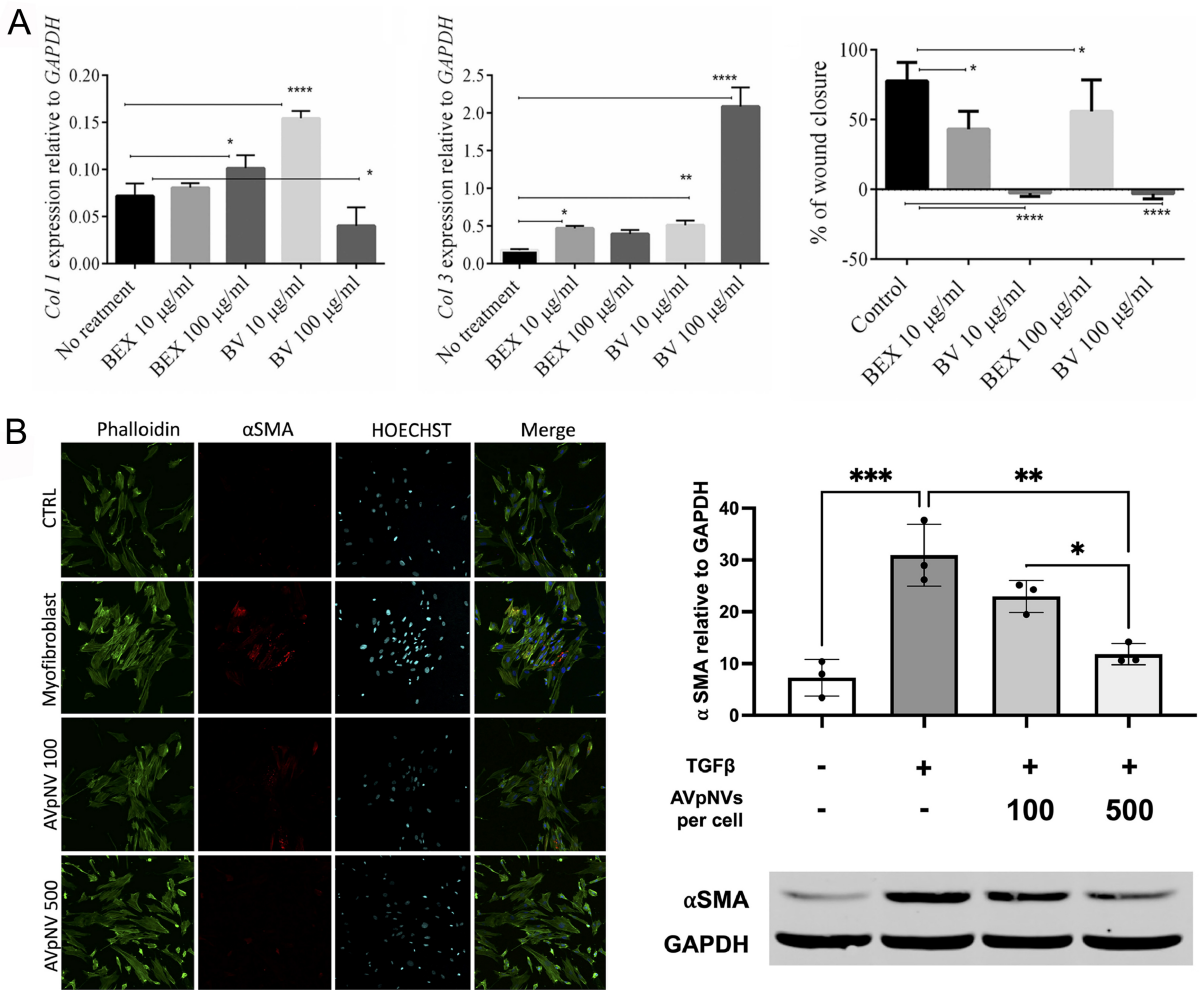
Applications of PDEVs in anti-scarring. (A) Effects of BV or BEX treatment on the expression of collagen I/III genes and *the* migratory capacity of fibroblasts. ^*^P ≤ 0.05; ^**^P ≤ 0.01; ^****^P ≤ 0.0001. This figure is quoted with permission from Mahdipour *et al*.^[23]^; (B) Effect of AVpNVs on myofibroblast differentiation. ^*^P < 0.05; ^**^P < 0.01; ^***^P < 0.001. This figure is quoted with permission from Ramírez *et al*.^[24]^. PDEVs: Plant-derived extracellular vesicles; BV: beta vulgaris; BEX: beta vulgaris extract; AVpNVs: aloe vera-derived nanovesicles; α-SMA: α-smooth muscle actin; TGF-β: transforming growth factor-β.

As previously mentioned, regulating collagen synthesis is a critical determinant of scar formation, with the relative abundance of collagen subtypes influencing both scar structure and repair outcomes. Collagen I, a primary constituent of scar tissue, provides temporary mechanical strength to wound areas. In contrast, the finer architecture of collagen III contributes to skin softness and elasticity. Therefore, modulation of collagen proportions, particularly an increased collagen III/I ratio, has been shown to improve scar remodeling^[67]^. Mahdipour isolated exosomes from Beta vulgaris extract (BEX) and investigated their effects on the migration and gene expression profiles of dermal fibroblasts. Notably, the study revealed that BEX possessed anti-scarring ability by inhibiting fibroblast migration and increasing the ratio of collagen III to collagen I [Figure 2A]^[23]^. Furthermore, both the TGF-β1 signaling pathway and myofibroblasts play central roles in collagen synthesis and are key targets in anti-scar strategies. Ramírez *et al*. reported that *Aloe vera* peel-derived EVs (AVpNVs) inhibited TGF-β1-induced myofibroblast differentiation and reduced contractility, thereby attenuating scar formation in early wound healing [Figure 2B]^[24]^. In a clinical case, a 36-year-old patient exhibited improvement in facial scars following combined treatment with rose stem cell‐derived exosomes (ASCEplus Derma Signal Kit/SRLV, ExoCoBio Inc., Seoul, South Korea) and two sessions of Dermapen microneedling over 12 days^[25]^.

In summary, PDEVs show potential as a therapeutic strategy for promoting wound healing and preventing scar formation through their anti-inflammatory and collagen remodeling properties.

### Anti-aging

#### Skin aging process

**Figure 3:**
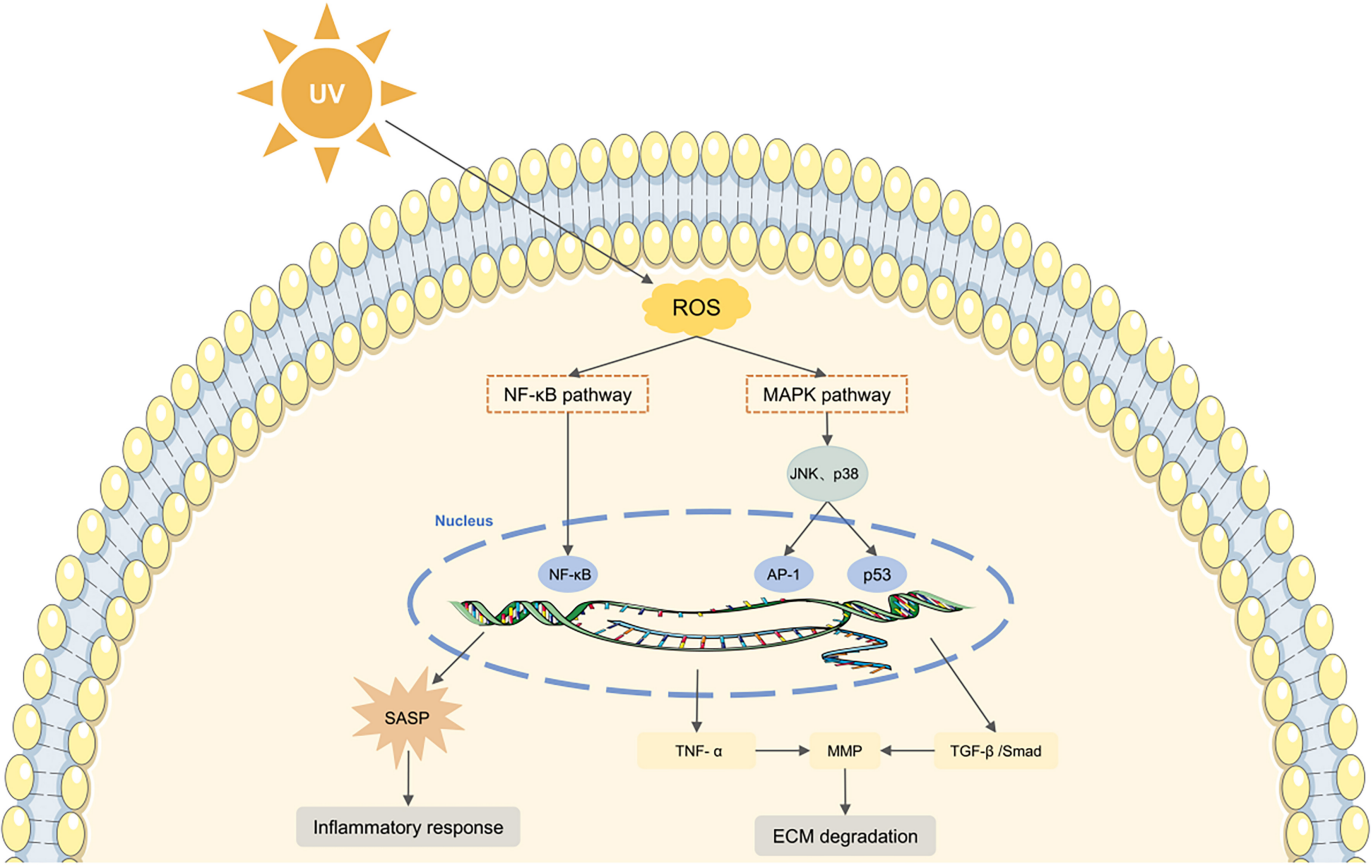
Schematic representation of ROS-mediated skin aging. UV-induced ROS modulate the MAPK and NF-κB signaling pathways, triggering inflammation and promoting ECM degradation, ultimately leading to skin aging. UV: Ultraviolet; ROS: reactive oxygen species; NF-κB: nuclear factor kappa B; SASP: senescence-associated secretory phenotype; MAPK: mitogen-activated protein kinase; JNK: c-Jun N-terminal kinase; AP-1: activating protein-1; TNF-α: tumor necrosis factor-α; MMP: matrix metalloproteinase; TGF-β: transforming growth factor-β; ECM: extracellular matrix.

Skin aging is influenced by both intrinsic biological processes and extrinsic environmental factors^[68]^. Intrinsic aging, which is genetically determined, manifests as a gradual decline in physiological function over time. In contrast, extrinsic aging primarily results from environmental exposure, with ultraviolet (UV) radiation being the predominant cause. This form of UV-induced aging is known as photoaging^[69]^. Photoaged skin is characterized by epidermal thickening, disorganized collagen, accumulation of abnormal elastic fibers, and alterations in cellular morphology^[70]^. A key mechanism in photoaging involves the accumulation of ROS, which induces DNA damage and oxidative stress, thereby accelerating photodamage [Figure 3]^[71]^.

Wrinkles and skin laxity are caused by progressive dermal atrophy, which is primarily driven by an imbalance between ECM synthesis and degradation^[72]^. Collagen, accounting for approximately 70% of the adult dermal ECM, plays a critical role in maintaining skin integrity and elasticity^[73]^. With aging, the proliferative capacity and synthetic activity of fibroblasts become significantly impaired, leading to an altered collagen I/III ratio and promoting extensive collagen fiber degradation and fragmentation^[74]^. Concurrently, UV-induced ROS enhance MMP-mediated collagen breakdown, without a compensatory increase in tissue inhibitors of metalloproteinases (TIMP)^[75]^. Dysregulation of the MMP/TIMP ratio accelerates collagen fragmentation, creating a self-perpetuating cycle of ECM deterioration and skin aging. In addition, ROS suppress TGF-β signaling, a key pathway for collagen synthesis. Collectively, these changes contribute to loss of skin firmness and ultimately lead to wrinkle formation.

Furthermore, inflammation is intimately linked to skin aging. During the aging process, fibroblasts progressively develop a senescence-associated secretory phenotype (SASP), characterized by sustained secretion of MMP and pro-inflammatory cytokines such as IL-1β, IL-6, IL-8, and TNF-α^[76]^. The SASP reinforces fibroblast senescence and local inflammation through autocrine signaling and also induces senescence in neighboring normal cells via paracrine mechanisms^[77]^. Therefore, inhibiting oxidative stress and inflammatory responses, alongside promoting collagen synthesis, is crucial for mitigating skin aging.

#### PDEVs inhibit skin aging

Studies have shown that EVs secreted by young cells alleviate cellular senescence in aged organisms by reducing oxidative stress and lipid peroxidation^[78]^. Separately, You *et al.* reported that EVs carrying collagen-encoding mRNA not only elevate collagen content but also enhance fibroblast proliferation^[79]^. Consequently, EVs have garnered research interest as a promising modality in cutaneous anti-aging strategies. The anti-aging effects of PDEVs have been extensively investigated and are potentially mediated through the inhibition of ROS generation, enhancement of antioxidant capacity, and promotion of collagen synthesis.

**Figure 4:**
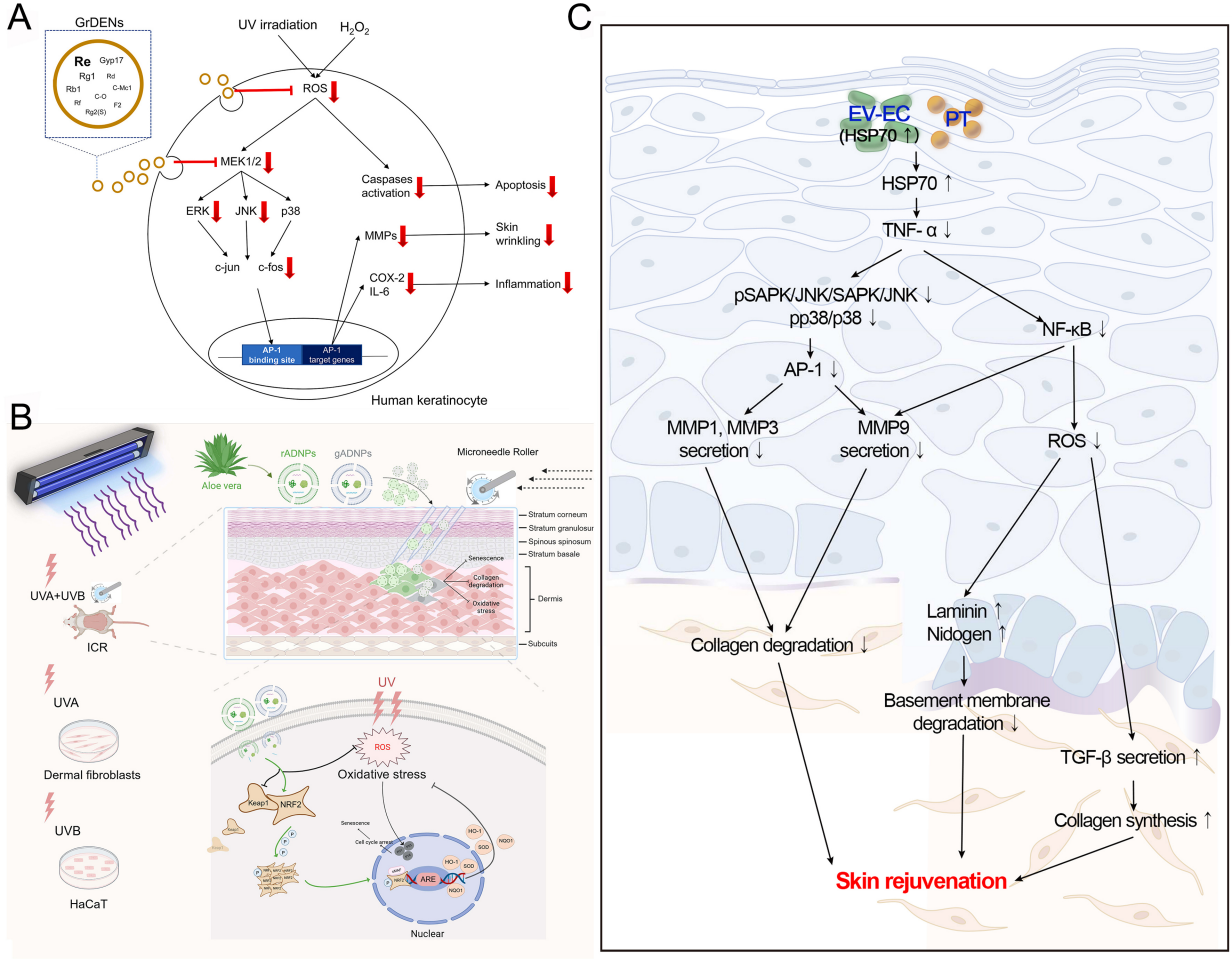
Mechanisms of some PDEVs against skin aging. (A) GrDENs. This figure is quoted with permission from Choi *et al*.^[26]^; (B) ADEVs. This figure is quoted with permission from Sun *et al*.^[28]^; (C) EV-EC. This figure is quoted with permission from Batsukh *et al*.^[31]^. PDEVs: Plant-derived extracellular vesicles; GrDENs: ginseng root-derived extracellular nanovesicles; ADEVs: aloe-derived extracellular vesicles; EV-EC: extracellular vesicles from epidermal cells; UV: ultraviolet; ROS: reactive oxygen species; ERK: extracellular signal-regulated kinase; JNK: c-Jun N-terminal kinase; MMP: matrix metalloproteinase; COX: cyclooxygenase; IL:interleukin; AP-1: activating protein-1; UVA: ultraviolet A; UVB: ultraviolet B; ICR: Institute of Cancer Research; HaCaT: human keratinocyte cell line; rADNPs: raw aloe-derived nanoplates; gADNPs: gel aloe-derived nanoplates; Keap1: Kelch-like ECH-associated protein 1; NRF2: nuclear factor erythroid 2-related factor 2; ARE: antioxidant response element; HO-1: heme oxygenase-1; SOD: superoxide dismutase; NQO1: NAD(P)H quinone oxidoreductase 1; HSP70: heat shock protein 70; PT: phlorotannin; TNF-α: tumor necrosis factor-α; SAPK: stress-activated protein kinase; NF-κB: nuclear factor kappa B; TGF-β: transforming growth factor-β.

PDEVs have been shown to reduce UV-induced ROS production. Choi *et al*. reported that ginseng root-derived exosome-like nanoparticles (GrDENs) inhibited ROS generation by suppressing AP-1 signaling, thereby protecting against UV-induced skin damage [Figure 4A]. Additionally, GrDENs downregulated the mRNA expression of aging-related genes (MMP-2 and MMP-3), pro-inflammatory genes (COX-2 and IL-6), and the cellular senescence marker p21^[26]^. Beyond scavenging ROS directly, PDEVs can attenuate skin aging by enhancing endogenous antioxidant defense systems. For example, López de Las Hazas *et al*. found that PDEVs transport various dietary polyphenols that have antioxidant effects^[80]^. Similarly, Logozzi *et al.* observed that nanovesicles from organically grown fruits and vegetables exhibited stronger antioxidant activity than those from conventional farming^[81]^. Further studies indicated that golden cherry (*Physalis minima*) exosomes possessed potential anti-aging effects due to their potent antioxidant properties^[27]^. The transcription factor nuclear factor erythroid 2-related factor 2 (Nrf2), a master regulator of antioxidant responses, activates genes encoding heme oxygenase-1, catalase, and superoxide dismutase (SOD), and plays a pivotal role in modulating aging^[82]^. Sun *et al*. demonstrated that *aloe vera*-derived EVs (ADEVs) mitigated skin photoaging by activating the Nrf2 pathway, which prevented UV-induced oxidative stress and suppressed the expression of β-gal and SASP. In a photoaging mouse model (ICR), ADEVs reduced malondialdehyde (MDA) levels and enhanced SOD activity in skin tissue, thereby delaying photoaging [Figure 4B]^[28]^.

As a reduction in collagen is a primary factor in skin aging, promoting its production constitutes a key therapeutic strategy. Katayama *et al*. reported that EVs from glucoraphanin-enriched kale activated the TGF-β signaling pathway to stimulate collagen synthesis, thereby attenuating skin aging^[29]^. Meanwhile, PDEVs inhibit collagen degradation by reducing MMP expression. For example, ginseng exosomes and green tea exosomes significantly downregulate MMP levels in skin cells^[30]^. Batsukh *et al*. demonstrated that EVs from *Ecklonia cava* (EV-EC) carry heat shock protein 70 (HSP70), which inhibits MMP production via suppression of the MAPK and NF-κB signaling pathways^[31]^. In addition, these EVs promoted collagen accumulation and improved skin elasticity in an aged mouse model [Figure 4C]^[31]^. Similarly, exosome-like nanovesicles from *Olea europaea* leaves downregulated NF-κB signaling, effectively reducing UV-induced skin damage and cellular senescence^[32]^. In another study, Trentini *et al*. reported that apple-derived EVs enhanced collagen synthesis while suppressing the production of MMP (MMP1, MMP8, and MMP9), collectively improving skin aging^[33]^. Consistently, extracellular vesicle-like nanovesicles derived from *Polygonum multiflorum* alleviated UV-induced oxidative stress, suppressed MMP1 expression, and increased the collagen III/I ratio in a nude mouse photoaging model^[34]^. Furthermore, balloon flower root-derived EVs were shown not only to reduce MMP expression but also to inhibit ROS generation and pro-inflammatory cytokine production^[35]^. In conclusion, the anti-aging effects of PDEVs are mediated through the reduction of ROS, enhancement of antioxidant defenses, stimulation of collagen production, and inhibition of MMP activity.

### Anti-pigmentation

#### Pigmentation process

According to Grand View Research, the global market for skin lightening products is expected to expand at a compound annual growth rate (CAGR) of 5.5% from 2022 to 2030, reaching an estimated value of US$16.14 billion by 2030^[83]^. This growth highlights sustained consumer demand for treatments targeting hyperpigmentation. Under physiological conditions, melanocytes synthesize melanin to shield the skin from damage caused by UV radiation^[84]^. However, overproduction of melanin can lead to skin problems such as freckles, melasma, and age spots^[85]^. Although benign, these conditions may adversely affect psychological well-being. Therefore, effective strategies to reduce hyperpigmentation and achieve a more even skin tone are essential, with melanogenesis regulation representing a key therapeutic target.

**Figure 5:**
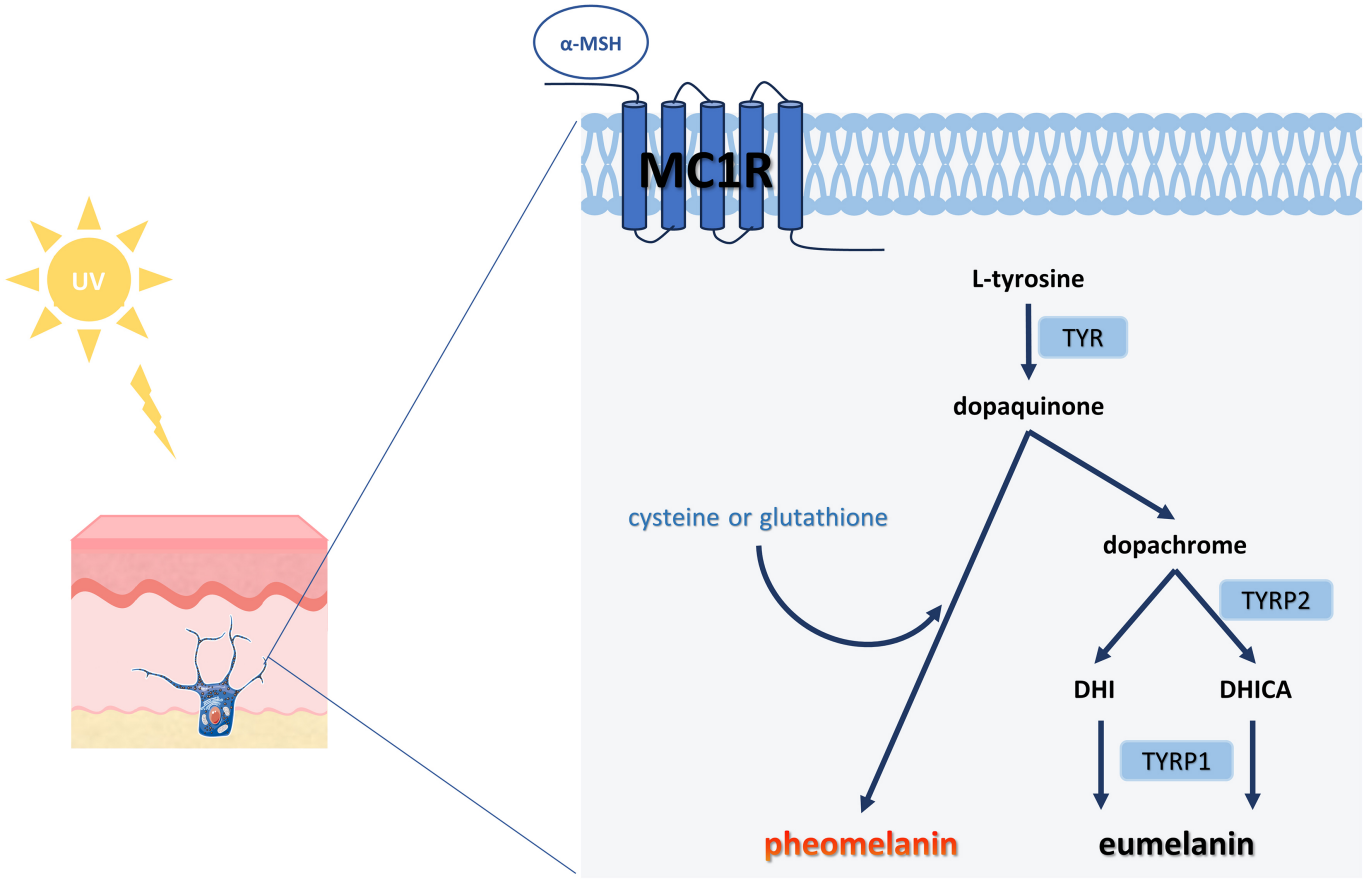
Schematic diagram of melanin production. The initial stage of melanogenesis is the production of dopaquinone from L-tyrosine, catalyzed by the rate-limiting enzyme TYR. Subsequently, dopaquinone undergoes a series of redox reactions to produce dopachrome, which forms both DHI and DHICA, eventually yielding brown-black eumelanin. Notably, if cysteine or glutathione is present, the dopaquinone reacts with it to ultimately produce the reddish-yellow pheomelanin^[86]^. TYR: Tyrosinase; DHI: 5,6-dihydroxyindole; DHICA: 5,6-dihydroxyindole-2-carboxylic acid; UV: ultraviolet; α-MSH: α-melanocyte-stimulating hormone; MC1R: melanocortin 1 receptor; TYRP1: tyrosinase-related protein 1; TYRP2: tyrosinase-related protein 2.

Melanogenesis entails a series of complex biochemical reactions mediated by multiple enzymes. Upon UV stimulation of the skin, keratinocytes secrete α-melanocyte-stimulating hormone (α-MSH), which binds to the melanocortin 1 receptor (MC1R) on melanocytes and activates cyclic adenosine monophosphate (cAMP) cascades, thereby promoting melanin synthesis [Figure 5]^[86,87]^. This process is further modulated by several signaling pathways, including cAMP /protein kinase A, mitogen-activated protein kinase, Wnt/β-catenin, phosphatidylinositol 3-kinase/Akt, and stem cell factor/c-kit signaling pathways, all of which converge to activate microphthalmia-associated transcription factor (MITF) expression^[88]^. As a master regulator, MITF plays a central role in melanogenesis by binding to M-box motifs in promoter regions of genes encoding key enzymes such as tyrosinase (TYR), tyrosinase-related protein 1 (TYRP1), and tyrosinase-related protein 2 (TYRP2). Beyond melanin synthesis, MITF also regulates melanocyte development, proliferation, and survival^[89]^. Newly synthesized melanin is packaged into melanosomes, which are subsequently transferred to keratinocytes^[90]^. As the skin undergoes natural turnover, these melanin-containing keratinocytes migrate upward to the stratum corneum, where they become visible and contribute to skin pigmentation.

#### PDEVs reduce hyperpigmentation

Current cosmetic approaches to skin lightening primarily target melanogenesis through three mechanisms: (1) inhibition of melanin synthesis (e.g., arbutin, kojic acid, tranexamic acid)^[91]^; (2) blockade of melanin transport (e.g., niacinamide)^[92]^; and (3) acceleration of melanin metabolism and skin renewal (e.g., vitamin C, salicylic acid)^[93]^. Since melanocytes reside in the epidermal basal layer, effective depigmentation requires transdermal delivery of active ingredients to this site. However, most functional ingredients are highly hydrophilic and exhibit poor skin permeability, which limits the efficacy of many lightening products^[94]^. Techniques such as ultrasound, radiofrequency, lasers, chemical permeation enhancers, and microneedling have been developed to enhance delivery^[95]^. Nevertheless, these methods often require specialized equipment, involve high costs, or carry safety risks including skin irritation and barrier impairment^[96]^. The limitations of traditional treatments have created a need for safer and more effective hyperpigmentation interventions.

Emerging evidence indicates that PDEVs possess considerable potential for inhibiting melanogenesis. Kusnandar *et al*. isolated PDEVs from yam bean (*Pachyrhizus erosus*) and demonstrated their anti-melanogenic effect in a zebrafish model, highlighting their potential application in skin lightening^[36]^. The primary anti-pigmentation mechanism of PDEVs involves the modulation of MITF and subsequent downregulation of TYR, TYRP1, and TYRP2, ultimately suppressing melanin production [Table 3]. For instance, exosome-like nanoparticles derived from *Atractylodes lancea* and *Panax ginseng* were shown to reduce melanin content by downregulating these melanogenic enzymes^[37,38]^. Byun *et al*. further revealed that EC-derived EVs attenuated melanogenesis and improved basement membrane structure by inhibiting the thioredoxin-interacting protein (TXNIP)/nucleotide-binding oligomerization domain-like receptor family pyrin domain containing 3 (NLRP3)/IL-18 pathway, a key upstream regulator of MITF^[39]^. In addition, Lee *et al*. reported that *Dendropanax morbifera*-derived EVs reduced melanin levels by modulating the α-MSH-MC1R pathway to suppress MITF expression, outperforming the anti-pigmentation effects of arbutin in human epidermal models^[40]^. Collectively, these findings highlight the promise of PDEVs as innovative agents for skin lightening.

**Table 3:** The mechanism of PDEVs in inhibiting pigmentation

**Source of PDEVs**	**Model**	**Mechanism**	**Reference**
*Atractylodes lancea*	*In vivo*: B16F10 cells	↓ melanin content, MITF, TYR, TYRP1, DCT	[37]
*Panax ginseng*	*In vitro*: HEM cells	↓ melanin content, TYR, TYRP2, RAB27	[38]
*Ecklonia cava*	*In vitro*: HaCaT, HEM cells *In vivo*: HRM-2 mice	↓ MITF, TYR, TYRP1, TYRP2 ↑ skin lightness	[39]
Leaves and stems of *D. morbifera*	*In vitro*: B16BL6 melanoma cells	↓ melanin content, MITF, TYR, TYRP1, TYRP2	[40]
*C. fragile* and *Sargassum fusiforme*	*In vitro*: MNT-1 cells *ex vivo* study: Human skin tissue	↓ melanin content, MITF, TYR, TYRP1	[41]
*Centella asiatica*	*In vitro*: B16F10 melanoma cells	↓ melanin content, TYR	[42]
RSCE	*In vitro*: B16F10 melanoma cells	↓ melanin content	[43]

↑ Represents upregulation; ↓ represents downregulation. PDEVs: Plant-derived extracellular vesicles; HEM: human epidermal melanocytes; MITF: microphthalmia-associated transcription factor; TYR: tyrosinase; TYRP1: tyrosinase-related protein 1; DCT: dopachrome tautomerase; TYRP2: tyrosinase-related protein 2; RAB27: ras-related protein 27; HaCaT: human keratinocyte cell line; HRM-2: hairless mice strain; MNT-1: a human melanoma cell line; RSCE: *Rosa damascena* stem cell extract; *C. fragile*: *Codium fragile*; *D. morbifera*: *Dendropanax morbifera*.

## THE CHALLENGES AND PROSPECTS OF PDEVS IN CLINICAL TRANSLATION

### PDEVs in clinical studies

Despite promising preclinical data on PDEVs in recent years, clinical investigations of their cutaneous applications remain relatively scarce. To date, only one registered clinical trial evaluating PDEVs has been identified on ClinicalTrials.gov: “Evaluation of Effects on Skin Quality of a Centella Asiatica Extracellular Vesicle-based Skin Care Formulation” (NCT06850935). This study assessed the impact of a *Centella asiatica* extracellular vesicle-based formulation on facial aesthetics and skin parameters in healthy volunteers. In a separate clinical investigation, Proietti *et al.* reported that combined treatment with microneedling and EVs derived from RSCE in 20 melasma patients resulted in significant improvement in the modified Melasma Area and Severity Index (mMASI) for 90% of participants. Notably, no severe adverse effects or post-inflammatory hyperpigmentation were observed, and all subjects reported excellent treatment tolerability^[97]^.

### Challenges in the application of PDEVs

In summary, while PDEVs show promise in facial aesthetics, current research remains preliminary, and industrial-scale manufacturing faces substantial challenges. To facilitate clinical translation, this review discusses and offers insights into future research directions.

First, the isolation and purification of PDEVs require standardized protocols to ensure reproducibility and scalability. Although multiple methods are available for PDEV extraction, current techniques are often limited by contamination risks, time-consuming procedures, or high costs to varying degrees [Table 4]^[98-103]^. Addressing these challenges calls for the development of unified industrial standards to improve the purity and yield of PDEVs. Membrane-based separation techniques, such as tangential flow filtration (TFF) and ionic membranes, offer advantages for scaling up due to operational flexibility and lower dependence on specialized equipment^[104]^. For example, Haraszti *et al*. observed a sevenfold increase in exosome yield using TFF compared to differential ultracentrifugation^[105]^. Similarly, Garcia *et al*. achieved large-scale production of functional, high-quality EVs from mesenchymal stromal cells using a hollow fiber bioreactor system^[106]^.

**Table 4:** Comparison of the common EV isolation methods

**Isolation methods**	**Advantages**	**Disadvantages**	**Reference**
Ultracentrifugation	The gold standard method; simple operation	Requires expensive equipment; high cost; time-consuming	[98]
Ultrafiltration	Rapid; does not require expensive equipment	Clogging of EVs, content reduction due to membrane adsorption	[99]
SEC	High purity; good reproducibility; preserves original exosomes structure	Time-consuming; limited sample volume; requires relatively expensive equipment	[100]
Immunoaffinity capture	High specificity and purity	Irreversible specific binding; affects subsequent experiments; not suitable for large-scale use	[101]
Polymer precipitation	Simple operation; used in large-scale processing	Low purity; presence of co-precipitates	[102]
Microfluidics-based isolation techniques	High purity and portability; rapid processing	Requires method standardization; low isolation capacity	[103]

EV: Extracellular vesicle; SEC: size exclusion chromatography.

Second, the identification of PDEVs presents a significant challenge. Current characterization methods primarily rely on physical properties such as particle morphology, size distribution, and zeta potential. However, due to the vast diversity of plant species, identifying universal biomarkers for PDEVs remains difficult. The Minimal Information for Studies of Extracellular Vesicles (MISEV2023), as updated by the International Society for Extracellular Vesicles (ISEV), designates CD9, CD63, and CD81 as characteristic markers of EV biogenesis. Unfortunately, these tetraspanins are not applicable to PDEVs. Although HSP70 has been detected in some PDEVs, specific and consistent protein markers for most PDEVs remain elusive^[107]^. Therefore, comprehensive profiling of PDEV cargo, such as lipids, transmembrane proteins, non-coding RNAs, and secondary metabolites, is urgently needed to establish reliable, plant-specific biomarkers.

Furthermore, the multifunctional nature of PDEVs, which is linked to their diverse cargo composition, underscores their therapeutic potential for various human diseases. Nevertheless, clinical translation faces practical barriers. For instance, PDEVs are unstable at room temperature and typically require storage at -80 °C. However, prolonged cryopreservation can reduce both the concentration and purity of PDEVs while increasing transportation costs, thereby hindering their commercial scalability^[108]^. Lyophilization offers a practical alternative to liquid storage. It is noteworthy that most PDEVs are stored in phosphate-buffered saline (PBS), but during freezing, the crystallization of certain components in PBS can lead to significant pH shifts that are detrimental to vesicle integrity. Therefore, the addition of cryoprotectants such as mannitol, alginate, or sucrose is often necessary^[109,110]^.

Finally, as an emerging therapeutic modality, PDEVs, like other biologic agents, require standardized quality control protocols. However, international regulatory harmonization is currently lacking. Hence, establishing unified guidelines for their isolation, purification, characterization, and quality control is critical to translate PDEV-based applications from laboratory research to clinical practice, necessitating global collaborative efforts. Currently, no established concentration ranges exist for PDEVs in facial aesthetics. Although existing studies generally support the safety profile of PDEVs, determining precise safety thresholds remains essential to minimize potential risks. Furthermore, since most studies on cosmetically relevant PDEVs have been conducted in animal models, their clinical efficacy may differ in humans due to interspecies variations in skin biology. This necessitates expanded clinical trials to verify their effects. Notably, the sourcing of PDEVs for cosmetic use requires rigorous scrutiny. EVs derived from conventionally farmed plants may retain pesticide residues, which could concentrate during isolation and purification, potentially introducing toxicity risks rather than conferring therapeutic benefits. Therefore, sourcing from organically cultivated plants is recommended to ensure safety and efficacy. Beyond origin, combining EVs from diverse plant species may yield optimized formulations tailored to specific therapeutic indications^[111]^. Additionally, the quality of PDEVs is influenced by natural variables such as geographical origin, seasonal variations, and climatic conditions during plant growth^[112]^. These factors challenge batch-to-batch consistency, thus necessitating strict quality control of raw plant materials to ensure experimental reproducibility and product reliability. In summary, considerable progress is required before PDEVs can be formally integrated into facial aesthetics. Nevertheless, their considerable therapeutic potential warrants continued investigation and development.

## CONCLUSION

Numerous studies have demonstrated that PDEVs have significant effects on anti-scarring, anti-aging, and anti-pigmentation, primarily *in vitro*. PDEVs hold broad applicability in facial aesthetics due to their nanoscale dimensions, efficient transdermal delivery, low immunogenicity, and relatively low cost. However, realizing their clinical value necessitates rigorous scientific validation and robust regulatory oversight. Therefore, it is imperative to develop optimized methods for the isolation, purification, characterization, and stable storage of PDEVs, as well as to establish comprehensive quality standards and regulatory frameworks to facilitate their translation into facial aesthetics.

## DECLARATIONS

### Copyright

© The Author(s) 2025.
